# Host–Guest Chemistry in Boron Nitride Nanotubes: Interactions with Polyoxometalates and Mechanism of Encapsulation

**DOI:** 10.1021/jacs.2c10961

**Published:** 2022-12-31

**Authors:** Jack W. Jordan, Alexander I. Chernov, Graham A. Rance, E. Stephen Davies, Anabel E. Lanterna, Jesum Alves Fernandes, Alexander Grüneis, Quentin Ramasse, Graham N. Newton, Andrei N. Khlobystov

**Affiliations:** † School of Chemistry, University of Nottingham, University Park, Nottingham NG7 2RD, U.K.; ‡ II. Physikalisches Institut, Universität zu Köln, Zülpicher Strasse 77, Köln 50937, Germany; § Russian Quantum Center, Skolkovo Innovation City, Moscow 121205, Russia; ∥ Nanoscale & Microscale Research Centre, University of Nottingham, University Park, Nottingham NG7 2RD, U.K.; ⊥ SuperSTEM, Laboratory, Keckwick Lane, Daresbury WA4 4AD, U.K.; # School of Chemical and Process Engineering & School of Physics and Astronomy, University of Leeds, Leeds LS2 9JT, U.K.

## Abstract

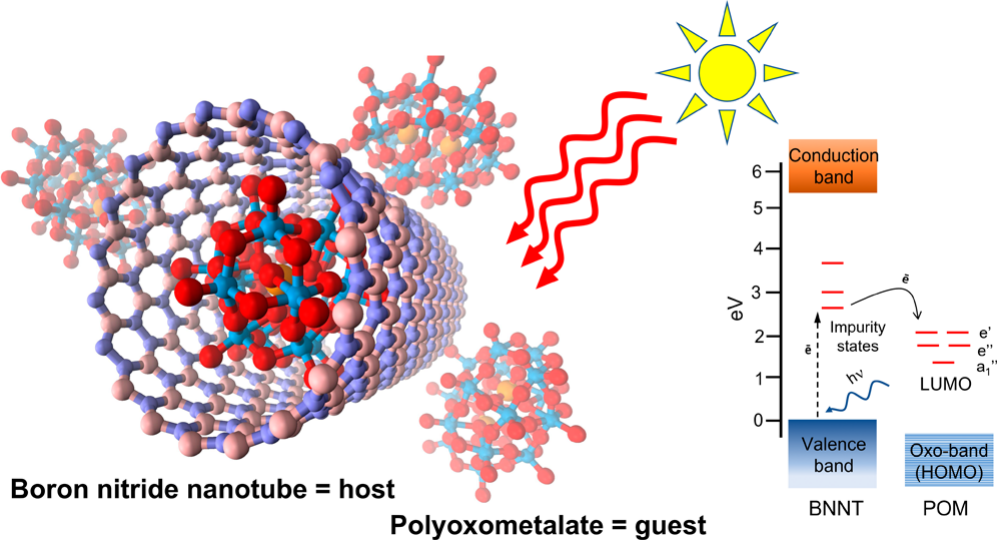

Boron nitride nanotubes (BNNTs) are an emerging class of molecular container offering new functionalities and possibilities for studying molecules at the nanoscale. Herein, BNNTs are demonstrated as highly effective nanocontainers for polyoxometalate (POM) molecules. The encapsulation of POMs within BNNTs occurs spontaneously at room temperature from an aqueous solution, leading to the self-assembly of a POM@BNNT host–guest system. Analysis of the interactions between the host-nanotube and guest-molecule indicate that Lewis acid–base interactions between W=O groups of the POM (base) and B-atoms of the BNNT lattice (acid) likely play a major role in driving POM encapsulation, with photoactivated electron transfer from BNNTs to POMs in solution also contributing to the process. The transparent nature of the BNNT nanocontainer allows extensive investigation of the guest-molecules by photoluminescence, Raman, UV–vis absorption, and EPR spectroscopies. These studies revealed considerable energy and electron transfer processes between BNNTs and POMs, likely mediated via defect energy states of the BNNTs and resulting in the quenching of BNNT photoluminescence at room temperature, the emergence of new photoluminescence emissions at cryogenic temperatures (<100 K), a photochromic response, and paramagnetic signals from guest-POMs. These phenomena offer a fresh perspective on host–guest interactions at the nanoscale and open pathways for harvesting the functional properties of these hybrid systems.

## Introduction

**Figure 1:**
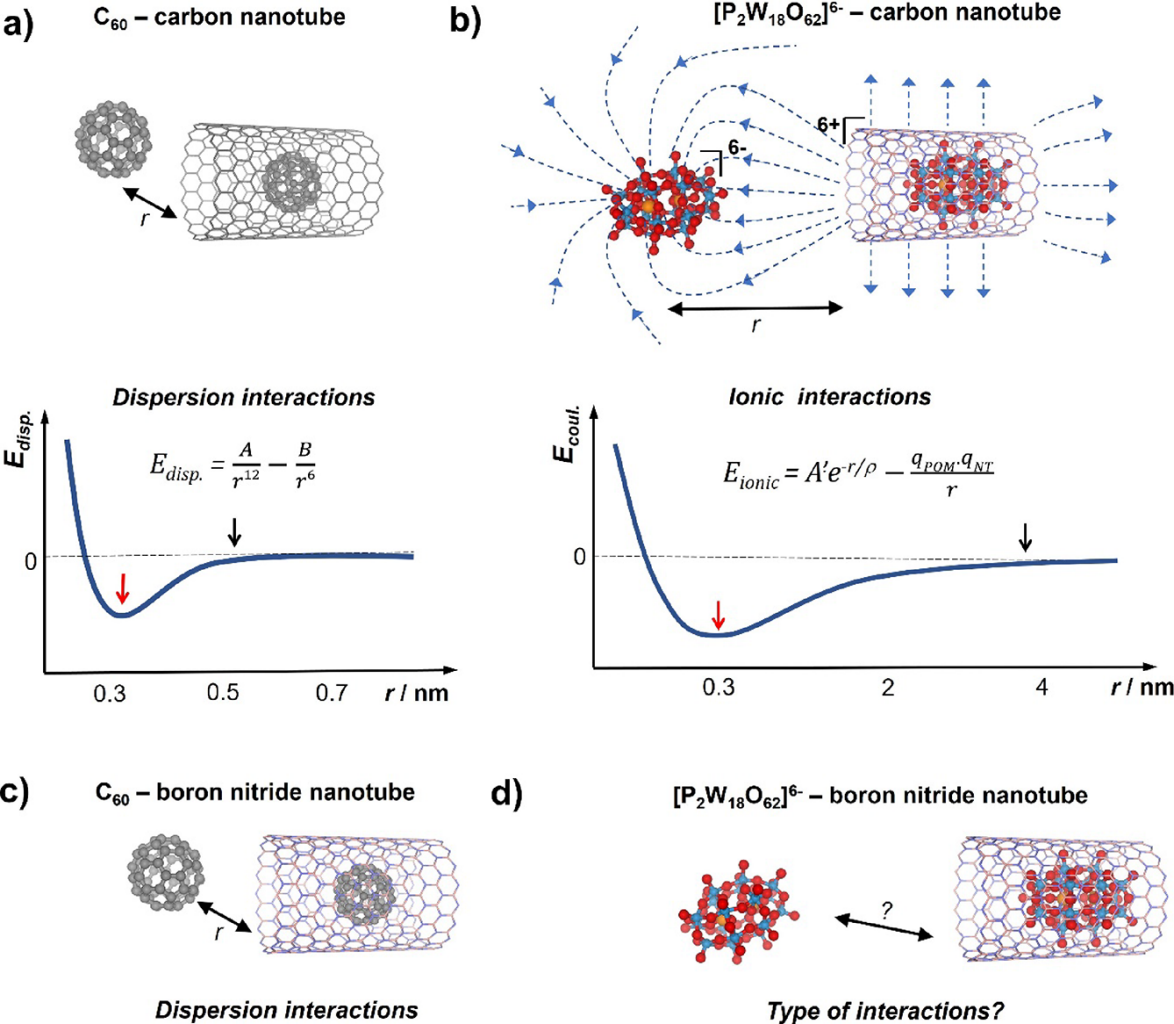
Encapsulation of charge-neutral guest-molecules, such as fullerenes, in carbon nanotubes (a) or boron nitride nanotubes (c) is driven by short-range dispersion interactions, decaying rapidly with distance *r*. In contrast, the encapsulation of charged redox-active molecules, such as POMs, in carbon nanotubes is facilitated by ionic interactions with the long-range attractive Coulombic term (b). Schematic plots of potential energy for dispersion (a) and ionic interactions (b) where *A*, *B, A′,* and ρ are constants, *q*_POM_ and *q*_NT_ are charges on the guest-molecule and host-nanotube, respectively (scales of the plots are approximate; red and black arrows indicate approximate distances corresponding to an equilibrium state and the onset of attractive interactions, respectively). In the case of the charged POM guest-molecules and boron nitride nanotubes (d), the nature of host–guest interactions remains unknown.

Nanotubes with two sets of dimensions, one at the nanoscale (diameter) and one macroscopic (length), provide a highly effective platform for bridging the molecular and macroscale worlds. The structure, dynamic behavior, and chemical reactions studied at the single-molecule level within these nano-test tubes can reveal fundamental chemical information that bulk measurements cannot access.^1^ On a practical level, nanotubes enable the functional properties of entrapped molecules to be effectively harnessed for catalysis and electrochemical and magnetic applications.^2^ A critical step in applications of these nanotube materials is the process of encapsulation of molecules within nanotubes, which requires a substantial thermodynamic driving force for the molecules to enter the nanoscale channel. For example, dispersion forces play a crucial role for the entrapment of fullerenes in carbon nanotubes (CNTs): being short-range, they rely on the complementary nature of the concave surface of the host-CNT and the convex surface of the guest-fullerene and can reach 280 kJ/mol for the ideal geometric match of C_60_ with ca. 1.3 nm diameter carbon nanotubes^3^ (Figure 1a). In the case of strong electron-donor^4^ or strong electron-acceptor guest-molecules,^5^ host-nanotubes and guest-molecules can exchange electron density, leading to electric charges that provide ionic interactions (with a Coulombic attractive term) that are significantly longer-range than dispersion forces (Figure 1b). In this context, polyoxometalate (POM) molecules, as strong electron acceptors, have recently been shown to spontaneously withdraw electrons from single-walled carbon nanotubes (SWNTs) at room temperature in water due to the POM LUMO being below the Fermi level of SWNTs.^5b^ The electron transfer is followed by a highly efficient entrapment of the negatively charged POM into the positively charged cavities of SWNTs (Figure 1b).

CNTs are excellent nano-test tubes, but they suffer from an important drawback—the lack of optical transparency associated with strong absorption of as-grown CNTs across the entire electromagnetic spectrum (the blackest material in the world^6^). While CNTs are an ideal nanocontainer for electrochemical^2^ and electron microscopy investigations of guest-molecules,^1b,7^ the opaque nature of CNTs creates significant hurdles for any spectroscopy methods to directly probe the guest-molecules. Recently, boron nitride nanotubes (BNNTs) entered the area of nanocontainers as a new type of host structure.^8^ Examples of encapsulated guests within BNNTs are still very scarce but include C_60_,^9,10^ metallic nanoparticles,^11−14^ and metal halides.^15^ The valence electron localization within the B–N bond means that the hexagonal boron nitride (hBN) lattice has far fewer free charge carriers than CNTs, meaning that BNNTs are wide-band gap semiconductors, with a band gap of approximately 5.5–6.5 eV (depending on the number of hBN layers).^16−19^ Unlike CNTs, the electronic band structure of BNNTs is independent of their chirality and diameter,^20^ meaning that all BNNTs are considered wide-band gap semiconductors. Consequently, carbon and boron nitride nanotubes have very different extinction coefficients of 45–55^21^ and 1.64 × 10^–4^ mL mg^–1^ cm^–1^ (at 204 nm),^22^ respectively, opening opportunities for probing electronic transitions of guest-molecules encapsulated within BNNTs with UV/visible light at least 270,000 times more effectively than in CNTs. Furthermore, the vibrational modes of guest-molecules are often obscured (“cloaked”) by the delocalized electron density of CNTs;^23^ as BNNTs have no delocalized charge carriers in the ground state, these vibrational modes are expected to become more accessible for molecules encapsulated within BNNTs. The transparent nature of BNNTs allows not only probing guest-molecules but also initiation of their photochemical reactions, as in the case of the polymerization of fullerene molecules in C_60_@BNNT triggered by visible light.^24^ Absorbing UV light, however, hBN and BNNTs generate short-lived excitons that may radiatively recombine,^25^ which may provide new mechanisms for the manipulation of guest-molecules with wavelengths that are not available with CNTs.

In this study, we assess BNNTs as nanocontainers of POM guest-molecules. A surprisingly effective spontaneous encapsulation of POMs into BNNTs yielded a POM@BNNT host–guest system, where the nature of molecule–nanotube interactions can be explored to a greater extent due to the transparent nature of the BNNT host, probed by a range of spectroscopy methods accessing their vibrational, electronic, and magnetic states (which are usually obscured in CNTs). Correlation of spectroscopy and microscopy data allowed us to identify key forces responsible for the interactions of BNNTs with guest-molecules, including those that govern the encapsulation mechanism, highlighting the importance of the electronic states associated with defects in BNNTs and the Lewis acidity of hBN lattice that enable BNNTs to behave as active nanocontainers.

## Experimental Section

### General

All common reagents and solvents were used as received from Sigma-Aldrich, Acros Organics, or Thermo Fisher. Boron nitride nanotubes (BNNT P1-Beta) were received from BNNT LLC. K_6_[P_2_W_18_O_62_] was prepared following a previously reported method.^26^

### Materials Preparation

BNNTs were cleaned and opened using base-catalyzed hydrolysis followed by high-temperature treatment and washing following the previously reported procedure.^10^ Purified BNNTs (10 mg) were heated at 200 °C for 1 h. Dried BNNTs were added to a 10 mM aqueous solution of K_6_[P_2_W_18_O_62_] (3 mL). The dispersion was agitated in an ultrasound bath for 5 min followed by stirring for 48 h at room temperature either under ambient light or in the dark. The pale blue (from ambient light experiments) or colorless solid (from dark experiments) was isolated by vacuum filtration using a PTFE membrane filter (pore size 0.2 μm) (12 mg).

### High-Resolution Transmission Electron Microscopy Imaging and Elemental Analysis

HRTEM images were acquired on a JEOL 2100+ LaB_6_ transmission electron microscope with an accelerating voltage of 100 kV. Samples were prepared by first dispersing them in isopropyl alcohol and then drop-casting them onto a copper grid coated with a “lacey” carbon film. All TEM images were processed using Gatan Digital Micrograph, and quoted distances were measured by drawing a line profile and measuring the image contrast intensity histogram. Energy dispersive X-ray (EDX) spectra were acquired during TEM imaging using an Oxford Instruments INCA X-ray microanalysis system.

### Aberration-Corrected Scanning Transmission Electron Microscopy

Additional imaging was carried out in a dedicated Nion UltraSTEM100 scanning transmission electron microscope (STEM) operated at the reduced acceleration voltage of 60 kV to minimize knock-on damage to the samples. The probe convergence was set at 32 mrad, with a probe current of 30 pA within an estimated 0.1 nm probe size. High-angle annular dark field images were recorded with a detector angular range of 90–185 mrad. Due to the highly dynamic behavior of the samples under the electron beam, series of fast images with 5 μs/pixel dwell time (1.3 s per frame) were recorded before aligning and averaging a small number of consecutive images after visual inspection for possible sample alteration.

### Variable-Excitation PL

Variable-excitation energy PL measurements were acquired with an Edinburgh Instruments FLS980 photoluminescence spectrometer at wavelengths of 250 and 275 nm. Confocal PL data was acquired with a Horiba-Jobin-Yvon iHR550 spectrometer with an excitation wavelength of 405 nm.

### Variable-Temperature Raman and PL (532 and 633 nm)

Spectra were collected across a range of temperatures from room temperature to 4 K using a Renishaw InVia Raman microscope and externally integrated Oxford instruments MicrostatHe cryostat mounted on a 3-axis coordinate stage. A 50× long-working distance microscope objective with an *N*_A_ of ∼0.4 and a focal distance of 20.5 mm for lasers with wavelength 532 and 633 nm was used, focusing the light inside the optical cryostat where the sample was mounted. For the measurements of Raman and PL response several gratings with the same Si detector have been used in order to cover different spectral ranges and provide suitable spectral resolution, in particular, 2400 g/mm for 532 nm wavelength and 1800 g/mm for 633 nm for Raman measurements. In order to detect broader PL lines and to measure larger spectral range in one accumulation, the 600 g/mm grating could be used. The laser power was set below 1 mW in each case as this was found to be the maximum power the material could tolerate before the onset of photothermal degradation. The focal spot diameter on the sample was around ∼1 μm.

### UV–Vis Absorption Spectroscopy

Solid state UV–vis spectroscopy was performed on an Agilent Cary 5000 UV–vis NIR spectrometer on neat sample powders. The data was acquired in reflectance mode, which was subsequently converted to a Kubelka–Munk function. Solution UV–vis spectroscopy was performed on a PerkinElmer Lambda 25 UV/Vis spectrometer in quartz cuvettes with the analyte dissolved in water.

### Illumination by Solar Simulator and Electron Paramagnetic Resonance Spectroscopy

Solid powder POM@BNNT was placed in a quartz tube and irradiated at room temperature in air and under Ar with a Newport Solar Simulator (1 sun) for 2 h. Color of the samples turned pale blue to a different degree, with a deeper color for POM@BNNT under argon. Electron paramagnetic resonance (EPR) measurements of irradiated samples were performed using Bruker EMX spectrometer at the X-band using Xenon software and detected a weak signal at *g* = 2.007.

## Results

**Figure 2:**
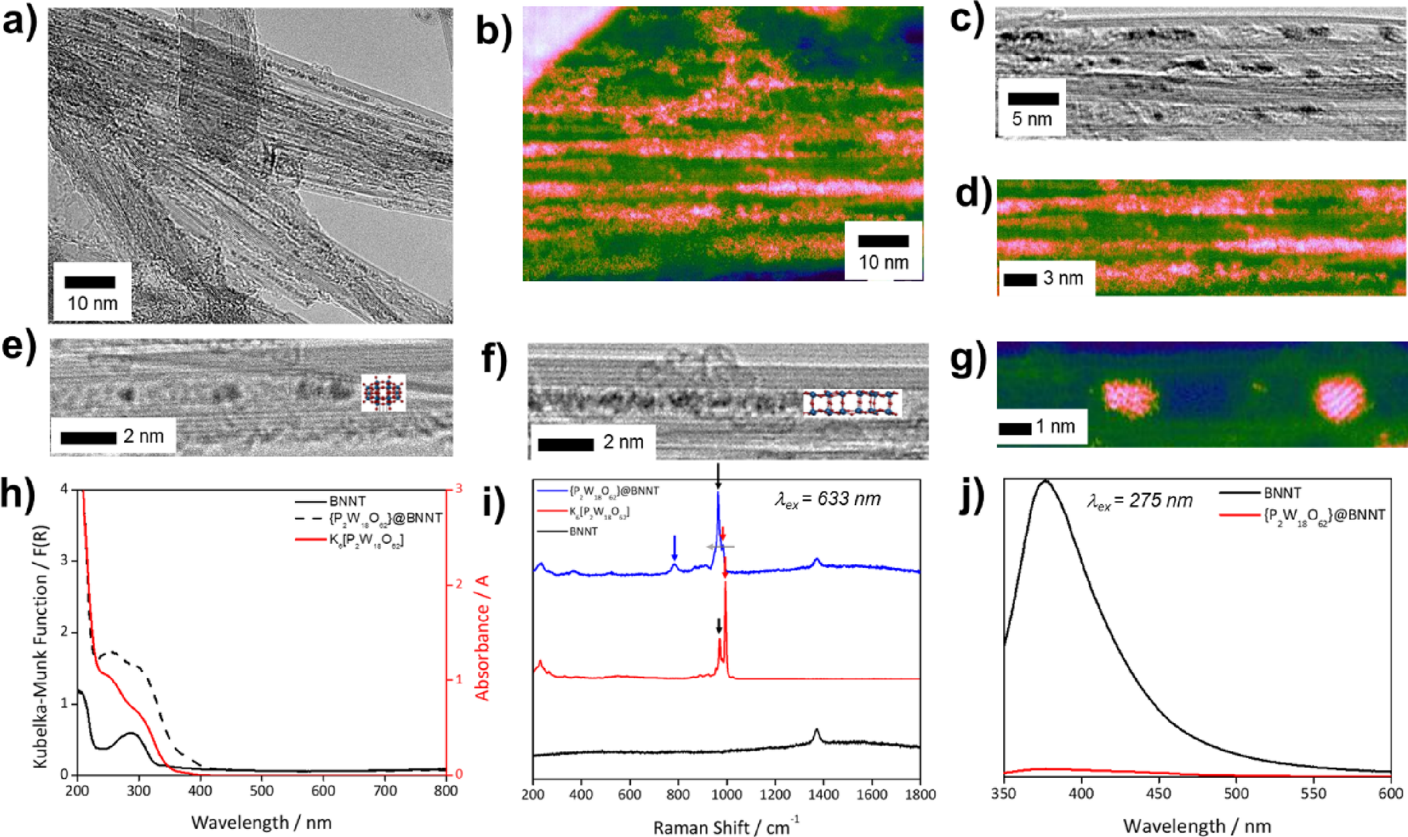
Large field of view 100 kV HRTEM (a) and 60 kV AC-STEM (b) imaging of POM@BNNT reveal that nanotube cavities are filled with high-contrast ∼1 nm species corresponding to polyoxometalate guest-molecules. High magnification imaging of individual nanotube bundles (c, d) and isolated nanotubes (e–g) indicates the sensitivity of POM molecules to electron beam irradiation, leading to polymerization to metal-oxide nanowires (f) (in AC-STEM images, orange contrast corresponds to W atoms and weak blue/green contrast corresponds to BNNT sidewalls). Solid-state UV–vis absorption spectra of POM@BNNT (dashed line), free POMs (red line, data gathered in solution), and empty BNNTs (black line) (h). Room-temperature 633 nm Raman spectra of POMs (red line), empty BNNTs (black line) and POM@BNNT (blue line), showing changes in the spectra upon POM encapsulation in nanotubes (i). Photoluminescence spectra of empty BNNTs centered at ∼370 nm (black line) becomes quenched upon encapsulation of POMs within the nanotubes (red line) (j) (excitation wavelength 275 nm; a similar effect was observed at other excitation wavelengths; SI file, Figures S3 and S4). Free POM molecules have no PL emission.

**Figure 3:**
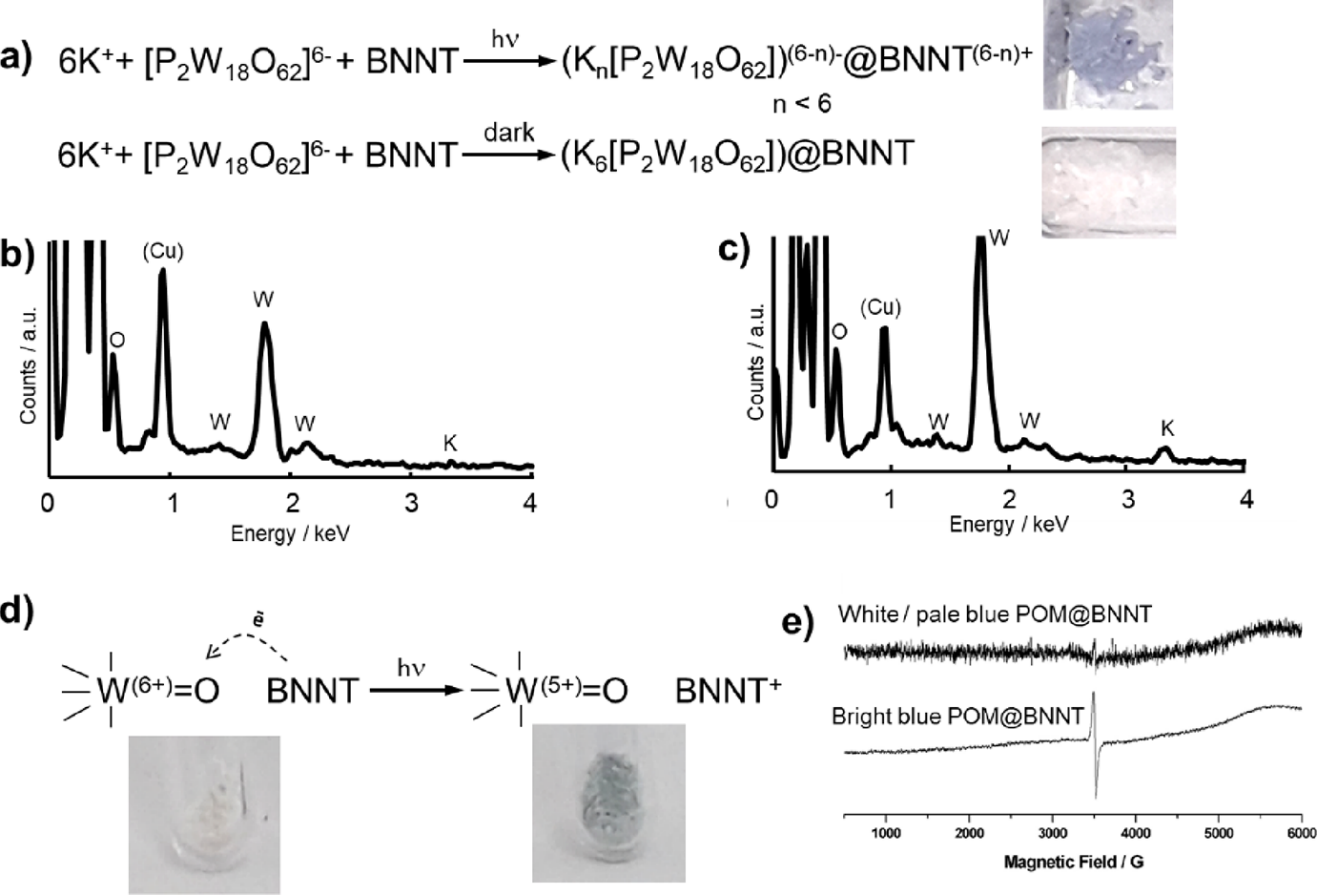
Balanced equation proposed for POM encapsulation under ambient light or dark conditions with corresponding photographs of POM@BNNT products formed (a). Non-stoichiometric content of K^+^, lower than required for balancing the charge of POM, was detected by EDX analysis performed during HRTEM imaging for small-bundle POM@BNNT of the material prepared under ambient conditions (b) and stoichiometry content of K^+^ commensurate with expectation was found in material prepared in the dark (c) (Cu peak from the sample holder). Colorless POM@BNNT turns to blue upon exposure to a solar light simulator over the course of an hour or ambient light over a period of several days, a color associated with the reduction of W centers. (d) A weak paramagnetic signal in EPR (e) can be detected for blue POM@BNNT samples.

In contrast to our previous reports with SWNTs,^5b,5c^ the POM solution did not change color instantaneously upon the addition of BNNTs under ambient light conditions, but a color change to blue was observed over the course of our experiment, albeit much less intense than that observed with CNTs. The blue color is due to the formation of so-called “heteropoly blues” and is indicative of reduced POMs present in solution.^27^ In a control experiment where BNNTs were added to a POM solution in the dark, the mixture remained colorless, indicating that light played a role in the POM reduction. TEM imaging revealed that in both cases, ca. 70% of BNNT cavities were filled with high-contrast species, the size of which was commensurate with the dimensions of POM molecules (ca. 1 nm) (Figure 2a–f). Local EDX analysis performed on individual BNNTs or bundles of BNNTs filled with POMs indicated that the presence of all elements was as expected (Figure 3b,c). Attempts to quantify the atomic composition of POM@BNNT by EDX was challenging due to the strong signals from B and N present in the host-nanotube. An atomic ratio W:O of 1:2.8 was observed, consistent with the POM composition, albeit with a slightly lower O content (W:O of 1:3.4 is expected), likely due to ejection of O under e-beam irradiation as seen previously with POM@CNTs.^28^ Interestingly, the ratio of K:W, which is 1:3 for K_6_[P_2_W_18_O_62_], appeared to be non-stoichiometric with less K than required to achieve charge balancing, particularly in the POM@BNNT material prepared under ambient light conditions (K:W ≈ 0.7:3). For the control sample prepared in the dark, a K:W ratio more consistent with the molecule’s stoichiometry was observed (K:W ≈ 0.9:3). Under the 100 keV e-beam of HRTEM and 60 keV e-beam of AC-STEM, guest-POM molecules within BNNTs appeared to be highly dynamic, changing their positions and orientations and rapidly polymerizing under the influence of the electron beam (Figure 2f), similar to the processes described for POMs in carbon nanotubes^28^ but with faster rates, making atomically resolved imaging of the guest-molecules within BNNTs challenging. Nevertheless, the atomic positions of W were observed in AC-STEM images where the molecules remained motionless for the duration of the scan (13 s per frame, with up to 2–3 consecutive frames displaying minimal atomic reconfiguration) (Figure 2g).

Our variable-excitation room temperature PL measurements (Figure 2j and Figures S3 and S4, SI file) confirm the existence of three energy levels at ∼2.00, 2.48, and 3.36 eV in empty BNNTs. The intensity of these PL emissions was quenched in POM@BNNT relative to the empty BNNTs at all excitation wavelengths. A significant color change from colorless to blue upon illumination of POM@BNNT was also observed (Figure S5, SI file). The optically transparent nature of the BNNTs allowed detailed spectroscopic analysis of the guest-POMs encapsulated within them. Solid state UV–vis spectroscopy measurements indicated distinct absorption bands of empty BNNTs and free POMs in the UV region, with the spectrum of POM@BNNT forming a superposition of the two (Figure 2h).

**Figure 4:**
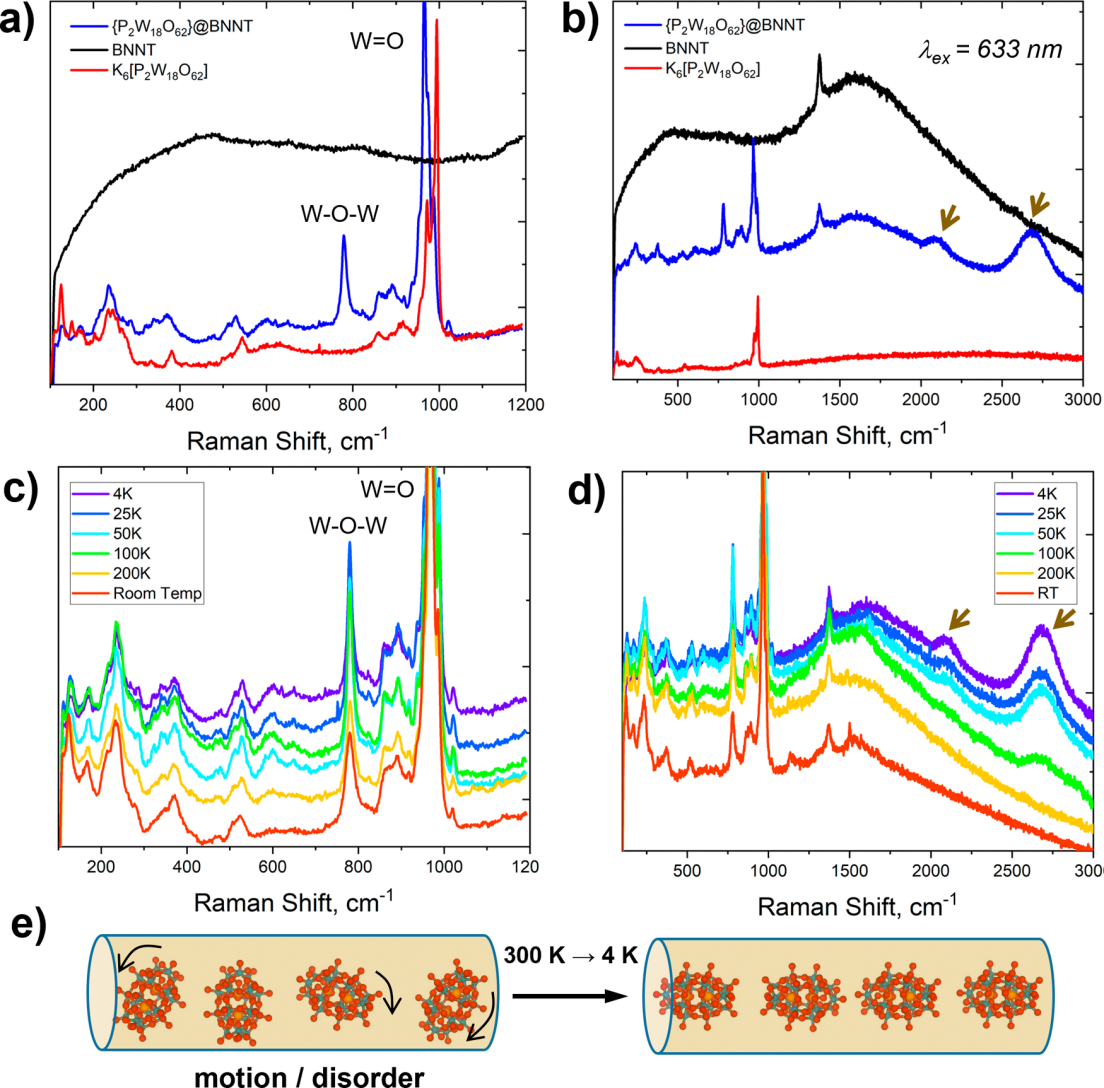
Comparison of cryo-Raman spectra (*T* = 4 K; excitation wavelength 633 nm) of POMs (red line); BNNTs (black line) and POM@BNNT (blue line) indicates changes in vibrations of W=O and W–O–W bonds in POM guest-molecules upon encapsulation (a) and new bands that emerge only in POM@BNNT system attributed to photoluminescence processes (marked with arrows) (b). The changes in Raman spectra of POM@BNNT become more pronounced as the temperature decreases from 300 to 4 K (c, d) as a result of diminished molecular motion and ordering of guest-molecules in BNNT cavities (e).

Raman spectra (excitation laser 633 nm) of empty BNNTs exhibit the *E*_2g_ band of the h-BN lattice superimposed on a background of weak PL (Figure 2i). The POM@BNNT spectra showed new features, including the emergence of a band at 777 cm^–1^, inactive within the POM crystal and tentatively assigned as a W–O–W bend, and changes to the W=O stretches at 950–1000 cm^–1^, which showed different intensities and shifted positions in the Raman spectrum relative to that seen in the spectrum of the POM crystal (Figures 2i and 4a). As the sample temperature was varied from RT to 4 K, the POM vibrational bands became more prominent and two new bands at around 2100 and 2680 cm^–1^ started to emerge below 100 K (Figure 4b,c and Figure S2, SI file). These bands could not be assigned to any specific vibration, appeared to be significantly broader than typical Raman bands, and are absent in the 532 nm Raman spectrum (Figure S2, SI file). An increase in the absolute intensity of all Raman bands with decreasing temperature was also observed, consistent with changes in the relative populations of the ground and excited vibrational states with temperature. A small relative increase in the intensity of the new band at 777 cm^–1^ was observed with decreasing temperature. The two new bands emerging at temperatures below 100 K can be interpreted as PL emission at 730 nm (2100 cm^–1^, 1.70 eV) and 762 nm (2680 cm^–1^, 1.63 eV), which increase in intensity with decreasing temperature.

It was noted that the initially white or pale blue POM@BNNT powder became bright blue in the area illuminated by a laser during PL or Raman experiments (Figure S5, SI file). A long exposure (ca. 3 months) to ambient light also increased the intensity of the blue color of POM@BNNT. A similar color change was replicated for the powder of POM@BNNT within an EPR tube illuminated for 2 h with a solar simulator (1 sun intensity). A weak EPR signal at *g* = 2.007 appears to be associated with the blue coloration of the POM@BNNT samples (Figure 3e).

## Discussion

Our observations demonstrate that POM anions can effectively enter the BNNTs from aqueous solutions spontaneously under ambient conditions, as evident from HRTEM, AC-STEM, and EDX analysis (Figures 2 and 3). This result is unexpected because the highly charged, hydrophilic nature of [P_2_W_18_O_62_]^6–^ anions and the charge-neutral hydrophobic BNNT cavity are seemingly incompatible. Therefore, it is important to identify the driving forces and mechanism for this process using our observations combined with prior knowledge of POM interactions with carbon nanotubes.^1,2^ We address this challenge first by assessing the state of the guest-molecule within the BNNT cavity versus free POMs. In this context, the optical transparency of BNNTs (unlike CNTs) offers new opportunities for probing the vibrational, electronic, and optical states of the encapsulated molecules, which we utilize here to ascertain the nature of interactions between the guest-molecules and host-nanotubes.

### Probing Guest-Molecules inside the BNNT

The optical transparency of BNNTs across a wide range of wavelengths allows the investigation of guest-molecules using light. Absorption bands of POM guest-molecules confined within BNNTs appeared at ∼250 and ∼300 nm corresponding to ligand-to-metal charge transfer (LMCT) transitions from the oxo-groups (W=O) to the W^6+^*d^0^*-metal centers^29^ and remained unchanged compared to free molecules (Figure 2h). The overall absorption spectrum of POM@BNNT was a superposition of the spectra of the guest-molecules and host-nanotubes (at ∼210 nm; Figure 2h). Defect-free BNNTs are expected to be wide-band gap semiconductors (∼5.5 eV)^30^ similar to h-BN; however, during BNNT growth, structural defects within the lattice, such as vacancies or substitutional defects, are known to introduce additional local states between the conduction and valence bands (2.5–4.5 eV).^31^ In the case of BNNTs used in our experiments, variable-excitation PL measurements at room temperature (Figure 2j and Figures S2 and S4, SI file) confirmed the existence of three such energy levels at ∼2.00, 2.48, and 3.36 eV, the presence of which is important for the consideration of photoactivated electron transfer from BNNT to POM, as described below.

**Figure 5:**
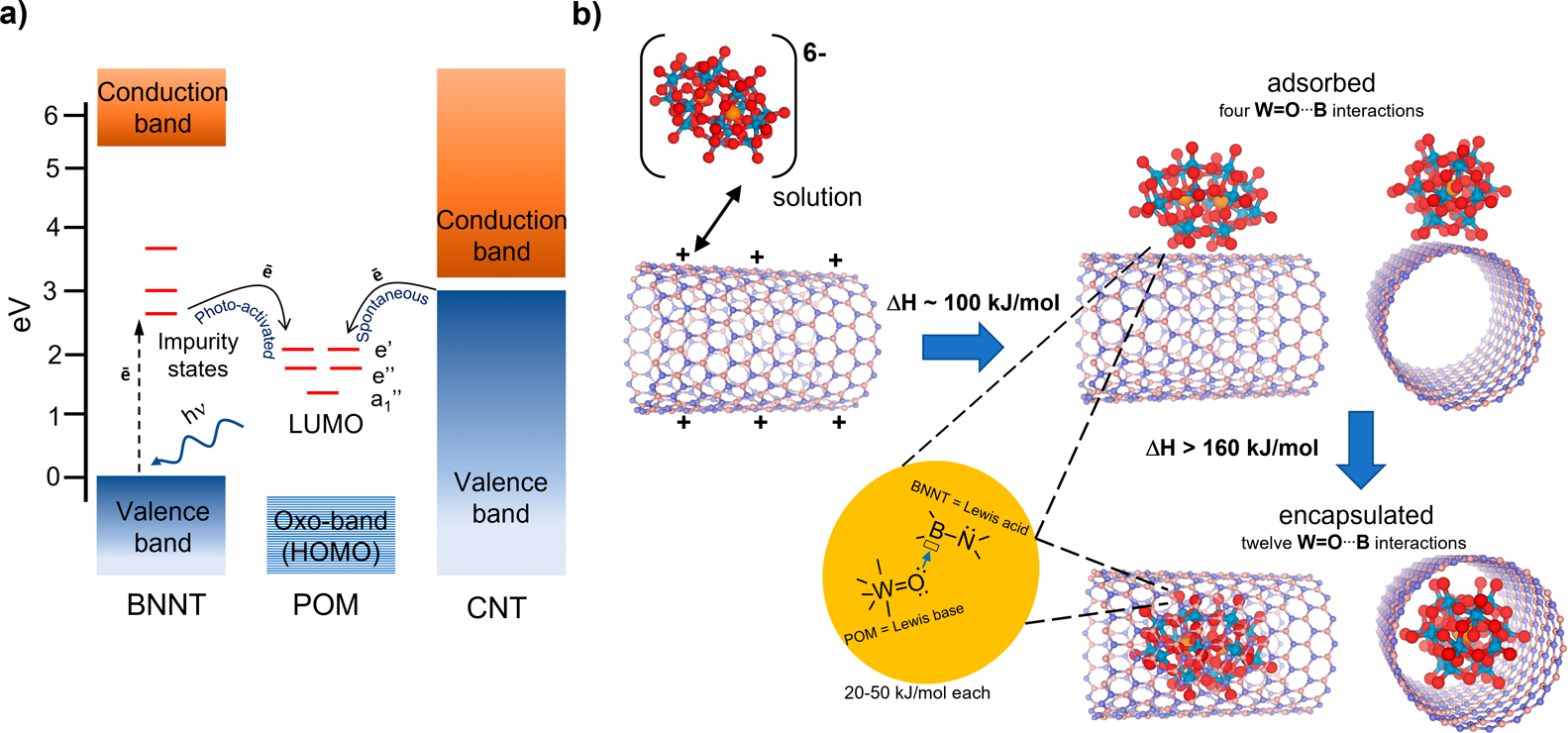
Proposed schematic energy diagram of [P_2_W_18_O_62_]^6–^ POM illustrating the alignment of the LUMO with electronic bands of carbon nanotubes (right) and boron nitride nanotubes (left) (a). While electron transfer from CNTs to POMs is spontaneous, this cannot occur for BNNTs without activation by photons. UV–vis and PL spectroscopy measurements indicate the presence of defect states inside the BNNT band gap accessible for electronic transitions in the visible range. This enables interactions of BNNTs with POM molecules, as evidenced by a color change of POM@BNNT in ambient light, and in cryo-Raman and PL measurements. The electronic transitions between BNNTs and POMs may contribute to the encapsulation process of the guest-molecules, driving the POM toward the BNNT in solution by Coulombic forces. However, short-range interactions, such as Lewis base–acid W=O···B bonding (lone pair on O donated to the empty *p_z_* orbital on B) are likely to be responsible for the encapsulation step due to an additive effect increasing from 4 bonds for POMs adsorbed on BNNTs to 12 bonds for POM@BNNT (b).

Vibrational modes of encapsulated molecules within the BNNTs were readily probed using Raman spectroscopy (Figure 2i) so that the entire Raman spectrum of guest-molecules could be measured in the POM@BNNT material, in contrast with carbon nanotubes that absorb strongly due to the resonant enhancement of surface plasmons at these wavelengths. While the photoluminescence of the BNNTs (originating from the defect states) is superimposed on the vibrational bands in the Raman spectra, it does not prohibit a detailed investigation of vibrations of the encapsulated guest-molecules. The state of the POMs within the nanotube is clearly different to that of free molecules. For example, the positions and intensities of the Raman bands corresponding to symmetric and antisymmetric W=O stretches are redshifted, suggesting O···B interactions of POM molecules with the concave side of BNNTs, where a lone pair on O is donated to the empty *p_z_* orbital on B (Figure 2i; scheme in Figure 5b). Furthermore, interactions of the guest-POMs with the host-BNNT interiors appeared to activate a vibration at ∼777 cm^–1^ (Raman silent in free POMs, but IR-active), which is assigned to a W–O–W antisymmetric stretch, as observed previously for POMs adsorbed on metal surfaces.^32^ Decreasing the temperature of POM@BNNT during Raman spectroscopy measurements from 300 to 4 K (Figure 4c,d) enhanced the signal-to-noise ratio as well as suppressed molecular motion such that non-covalent interactions between the POM and BNNT become stabilized, resulting in the band at 777 cm^–1^ becoming more prominent. The observed temperature dependence is likely a result of the reduced molecular motion of the POM molecules within the BNNT cavity at low temperature. It is known that guest-molecules within the nanotubes retain some degrees of rotational freedom (and in the case of sparsely filled nanotubes, translational freedom)^33^ due to the atomically smooth hexagonal lattice creating virtually no barriers for molecular motion at room temperature. As the temperature in Raman experiments decreased, molecular motion slowed down, allowing the molecules to relatively order (Figure 4e) and for host–guest interactions to manifest in the vibrational modes (Figure 4c).

### Electron Transfer between the POM and BNNT

Room-temperature PL measurements revealed significant quenching of the emission of nanotubes by the guest-molecules in POM@BNNT, indicating an effective electron transfer from photoexcited host-BNNTs to guest-POMs followed by non-radiative relaxation of the excitons (Figure 2j) as no new emission bands could be observed in the POM@BNNT material under the excitation wavelengths between 270 and 405 nm at 300 K (Figures S3 and S4, SI file). It is interesting to note that upon illumination with UV or visible light at room temperature, the color of POM@BNNT material turned from colorless to blue, indicating an electron transfer from photoexcited BNNTs to POMs was possible, causing partial reduction of the guest-molecules (Figure 3d). Weak paramagnetism seems to be associated with the blue coloration of POM@BNNT, as indicated by an EPR signal *g* = 2.007, suggesting the formation of free-radicals in the host–guest system during illumination (Figure 3e); however, it is not possible to establish the exact location of the paramagnetic centers in the system due to the weak EPR signal intensity.

In the Raman spectrum of POM@BNNT, two new bands at 2100 and 2680 cm^–1^ emerged as the temperature decreased below 100 K (Figure 4d and Figure S5, SI file), and they remain broader than any of the Raman bands belonging to BNNTs or POMs. These observations suggest a non-vibrational origin, which therefore can be considered as emissions at energies of 1.70 and 1.63 eV taking place in the POM@BNNT system. Electrons excited by 633 nm photons from the BNNT valence band to defect states are likely to transfer to the LUMO of the POMs (Figure 5a), which upon return back to the valence band of BNNTs may explain the emission observed at low temperature, superimposed onto the Raman vibrational spectrum POM@BNNT. The fact that these emission bands emerge only below 100 K indicates that non-radiative relaxation pathways that exist at room temperature become inhibited below 100 K. Using a shorter wavelength of 532 nm for Raman laser excitation, the two new emissions could not be observed in the POM@BNNT material at any temperature due to significant photoluminescence of BNNTs when excited by the shorter-wavelength laser as 532 nm allows access to higher density of defect states, resulting in more intense PL of the BNNTs themselves that obscures the POM LUMO-to-BNNT transitions (Figure S5, SI file).

Based on the sum of experimental evidence from PL, UV–vis absorption, Raman, and cryo-Raman observations, the following energy diagram can be proposed, illustrating photoactivated host–guest interactions in the POM@BNNT system (Figure 5a). Electrons of the BNNT valence band photoexcited by visible light can be transferred to the LUMO of encapsulated POMs, directly or via BNNT defect states located within the band gap, which results in the blue coloration of the material (Figure 4) (W^6+^ → W^5+^ reduction) and quenching of the boron nitride nanotube emission in POM@BNNT at room temperature (Figure 2j). The photoexcited state of the POM@BNNT system is metastable as electrons in the LUMO of encapsulated POMs appear to return to the valence band of BNNTs, giving rise to emissions at 1.63 and 1.70 eV, which can be detected at cryogenic temperatures in Raman spectra at ∼2680 and 2100 cm^–1^, respectively (Figure 4d). In all cases, the defect states in BNNTs seem to play a key role by providing additional energy levels essential for communication between the host-nanotube with guest-molecules. Due to the insulating nature of BNNTs, the redox activity of the guest-POMs could not be probed with voltammetric techniques. Cyclic voltammograms of the POM@BNNT material returned a purely capacitive response (Figure S6, SI).

### Driving Forces and Mechanism of POM Encapsulation in BNNT

The low-lying energy states in nanotubes filled with electrons are of paramount importance for encapsulation of POMs in carbon nanotubes as electron transfer from CNTs to POMs is followed by Coulombic attraction of negatively charged guest-molecules into positively charged host-nanotubes (Figure 1b).^2a,5b,5c,29^ However, our measurements have demonstrated that such states are absent in BNNTs, which possess no accessible electrons available for POM reduction (Figure 5a). Therefore, it is surprising that POM molecules enter BNNTs from water at room temperature so effectively in our experiments, as evidenced by extensive HRTEM and AC-STEM analysis of filled nanotubes (Figure 2a–g). We observe blue coloration of POMs with BNNTs in water occurring gradually (not as immediate and intense as in the case of CNTs under the same conditions^2a,5b,5c^), indicating that electron transfer from BNNTs to POMs may be possible under ambient conditions, even though the alignment of electronic bands of BNNTs and molecular orbitals of POMs is not favorable for this process to occur spontaneously (Figure 5a). Absorption and photoluminescence spectroscopy measurements for empty BNNTs provide evidence of additional electronic states at ∼2 eV, positioned within the band gap of the nanotube (Figure 2h,j), which can become populated upon excitation of BNNTs by visible light and then cascading electrons from BNNTs to the POM LUMO upon contact with the molecules. Such a process would enable the reduction of POMs (blue color) and positive charge accumulation on BNNTs that could provide a basis for the Coulombic host–guest attraction of POMs and BNNTs (Figure 5b). A control experiment in the absence of light showed that POMs can still be encapsulated within BNNTs without photoexcitation (and hence without electron transfer to POMs in solution) at room temperature in water with no significant decrease of the BNNT filling rate. Elemental analysis by EDX spectroscopy revealed a lower relative content of potassium in POM@BNNT prepared under ambient conditions (Figure 3b) than in the dark (Figure 3c). While the difference is subtle, it seems to indicate that at least some POM molecules can be encapsulated as anions ([P_2_W_18_O_62_]^6–^) without some or all charge-balancing cations (K^+^), like the encapsulation process in carbon nanotubes.^2,5b,5c^ However, when performing our encapsulation experiments in the dark, most POM guest-molecules entered the BNNTs as ion pairs (K_6_[P_2_W_18_O_62_]) (Figure 3) indicated by the 0.9:3 K:W atomic ratio.

These measurements suggest that the electron transfer and resulting Coulombic attraction between BNNTs and POMs appear to be coincidental rather than essential for POM encapsulation within BNNTs. The ubiquitous dispersion forces are unlikely to play a significant role due to low polarizability of POMs and BNNTs (both insulators with electrons locked in chemical bonds; for example, dispersion interactions between BNNTs and C_60_ result in only 125 kJ/mol energy gain,^34^ which should be expected to be even lower for POMs). Therefore, it is important to assess POMs and BNNTs for other possible interactions. Unlike the carbon lattice of CNTs, the boron nitride lattice of BNNTs contains sites with Lewis acidity and basicity, located on B and N atoms, respectively, which have the intrinsic capacity to accept or donate lone pairs from/to guest-molecules. The POM molecule [P_2_W_18_O_62_]^6–^ contains multiple lone pairs on W=O oxo-groups accessible for O···B bonding (while oxygen atoms in W–O–W groups possess a higher basicity, they are unlikely to engage in interactions with the concave side of BNNTs due to sterical hindrance), and W–O^–^ groups in a protonated form may engage in hydrogen bonding W–OH···N. Lewis base–acid O···B interactions are expected to be relatively weak in a region of 20 kJ/mol per bond or less,^35^ similar to H_2_O bonding mode to BNNT predicted theoretically;^36^ however, considering that up to 12 such interactions are possible between POMs and the concave surface of BNNTs, the total energy may add up to ca. 300 kJ/mol, which would be a substantial attractive force. Importantly, these interactions will be maximized when the POM is encapsulated within the nanotube as compared to POM on the nanotube external surface (a maximum of four O···B interactions are possible for POMs with the convex surface of BNNTs), thus making molecular encapsulation thermodynamically more favorable than adsorption of the molecules on the BNNT surface (Figure 5b). Raman spectroscopy measurements corroborate the existence of these interactions between the POMs and BNNTs (Figure 4). As POMs are dissolved in water during the entrapment process, K^+^ cations may exchange for protons, providing additional non-covalent interactions in a form of hydrogen bonding, W–OH···N, which are expected to be comparable to weak hydrogen bonds (∼15 kJ/mol)^37^ and may contribute to POM encapsulation. EDX analysis of POM@BNNT showed that K^+^ cations are present in almost stochiometric proportions for the K_6_[P_2_W_18_O_62_] formula, which suggests that W–OH are unlikely to play a role in POM@BNNT, and we have no spectroscopic evidence for this interaction. Overall, the interactions between the POMs and BNNTs appear to be a sum of different forces rather than a single well-defined force, with Lewis acid–base O···B interactions likely to contribute most to encapsulation, with secondary contributions from Columbic and dispersion forces, collectively providing conditions for the highly effective entrapment of POM guest-molecules in cavities of boron nitride nanotubes observed in our experiments. An elegant recent study demonstrated chaotropic effects in the process of polyoxometalate supramolecular assembly with cyclodextrin,^38^ which may additionally contribute to interactions between POMs and BNNTs.

## Conclusions

We have demonstrated boron nitride nanotubes as highly effective host structures for photo- and redox-active polyoxometalate guest-molecules. Encapsulation of POMs in BNNTs is spontaneous and irreversible at room temperature from aqueous solutions and does not require any activation, thus indicating a strong thermodynamic drive for the molecule’s entry into BNNTs. While there is an indication of electron transfer from the BNNTs to POMs under ambient conditions, it appears to be not the main driving force for the transport and encapsulation of the molecules (unlike CNTs). Instead, noncovalent Lewis base–acid O···B interactions between a lone pair of W=O oxo-group of the POM and the empty *p_z_*-orbital of boron atoms in BNNTs have been found to be important for stabilization of the encapsulated molecules, resulting in changes to the W=O vibrations of encapsulated POMs. The entropic effects of this encapsulation are currently under investigation in our laboratories. Our imaging and analysis of POM@BNNT systems emphasizes an important feature of BNNT hosts as compared to their much more widely used CNT counterparts—their high optical transparency allowed us to probe the guest-molecules with different types of radiation. This resulted in highly informative Raman, PL, UV–vis absorption, and EPR data not obscured by host-nanotubes, which offered essential details on host–guest interactions and identified forces responsible for encapsulation, revealing intriguing photo-redox behavior. The photo-stimulated electron and energy transfer processes discovered between the host-BNNTs and guest-POMs may provide a rich playground for new phenomena emerging as a result of nanoconfinement of photo/redox active molecules at the nanoscale. Photovoltaics, photocatalysis, sensing, and LED technologies may exploit the processes in host–guest systems driven by light. Indeed, our recent study has demonstrated a photochemically initiated polymerization of fullerene C_60_ in BNNTs,^24^ highlighting the potential of boron nitride nanotubes as nanoscale reactors for photochemical transformations.

## Supporting Information Available

Data including TEM, Raman spectroscopy, and PL spectroscopy (PDF)
