# Supplementary material for: Host–Guest Chemistry in Boron Nitride Nanotubes: Interactions with Polyoxometalates and Mechanism of Encapsulation
Source: J Am Chem Soc. 2022 Dec 31;145(2):1206–15. doi: 10.1021/jacs.2c10961 (PMC9853852; doi:10.1021/jacs.2c10961)
Supplement: Supplementary file 1 — ja2c10961_si_001.pdf [file ja2c10961_si_001.pdf]

Supporting Information

Host – Guest Chemistry in Boron Nitride Nanotubes:

Interactions with Polyoxometalates and Mechanism of Encapsulation

Jack W. Jordan,[1] Alexander I. Chernov,[2,3] Graham A. Rance,[1,4] E. Stephen Davies,[1] Anabel E. Lanterna,[1] Jesum Alves Fernandes,[1] Alexander Grüneis,[2] Quentin Ramasse,[5,6] Graham N. Newton,[1] Andrei N. Khlobystov*[1]

[1]School of Chemistry, University of Nottingham, University Park, Nottingham, NG7 2RD, U.K.

[2]II. Physikalisches Institut, Universität zu Köln, Zülpicher Strasse 77, 50937 Köln, Germany

[3]Russian Quantum Center, Skolkovo innovation city, 121205, Moscow, Russia

[4]Nanoscale & Microscale Research Centre, University of Nottingham, University Park, Nottingham, NG7 2RD, U.K.

[5]SuperSTEM, Laboratory, Keckwick Lane, Daresbury, WA4 4AD U.K.

[6]School of Chemical and Process Engineering & School of Physics and Astronomy, University of Leeds, Leeds LS2 9JT, U.K.

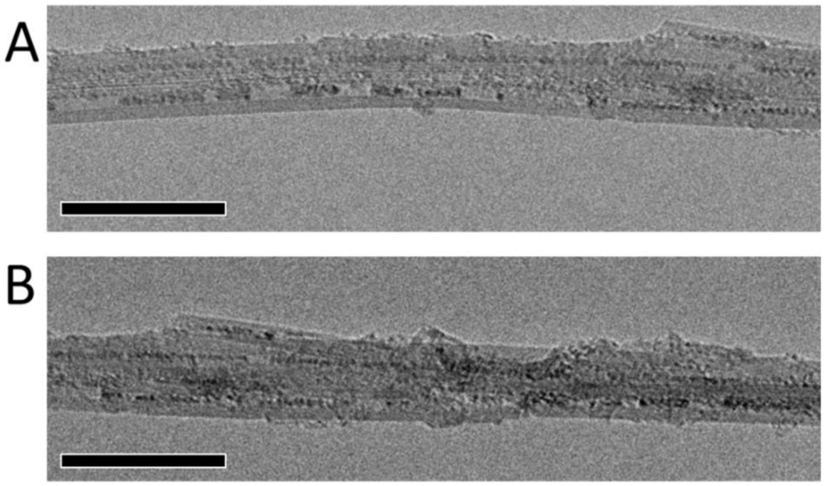

Figure S1. TEM images of POM@BNNT after (a) brief electron beam irradiation and (b) 30s electron beam irradiation. Images acquired at 80 kV. Scale bars are 20 nm.

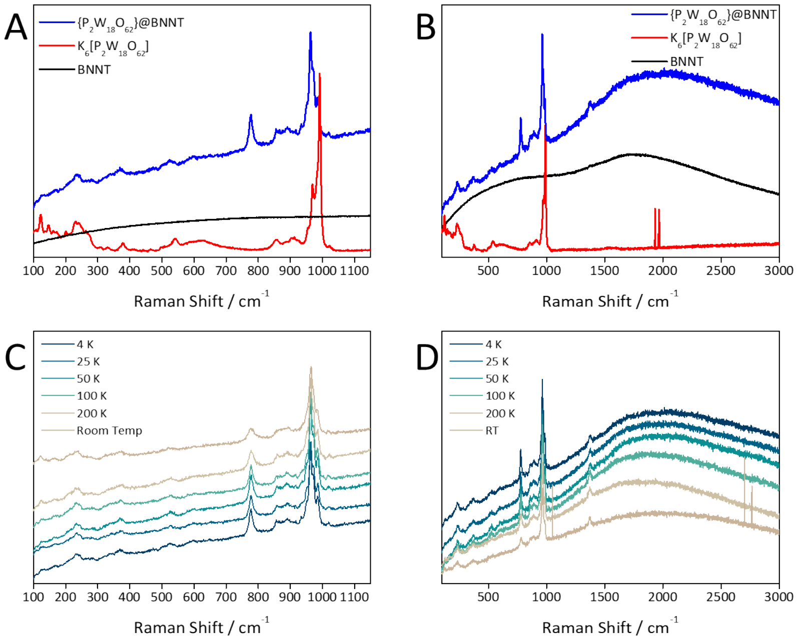

Figure S2. Raman spectra of BNNTs, $K_6[P_2W_{18}O_{62}]$ and $\{P_2W_{18}O_{62}\}$@BNNT at 4 K (A & B). Variable temperature Raman spectra of $\{P_2W_{18}O_{62}\}$@BNNT (C & D). Acquired with an excitation wavelength of 532 nm. Spikes in the spectra are cosmic interference. Intensities across the spectra are not comparable.

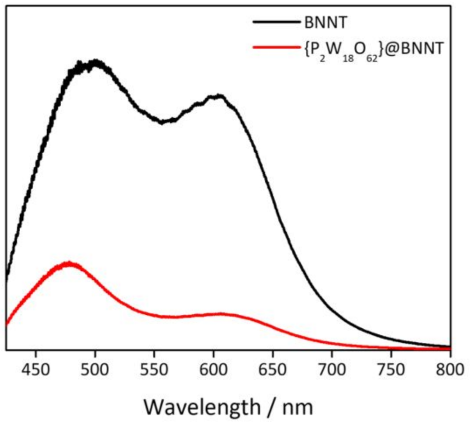

Figure S3. 405 nm PL spectra.

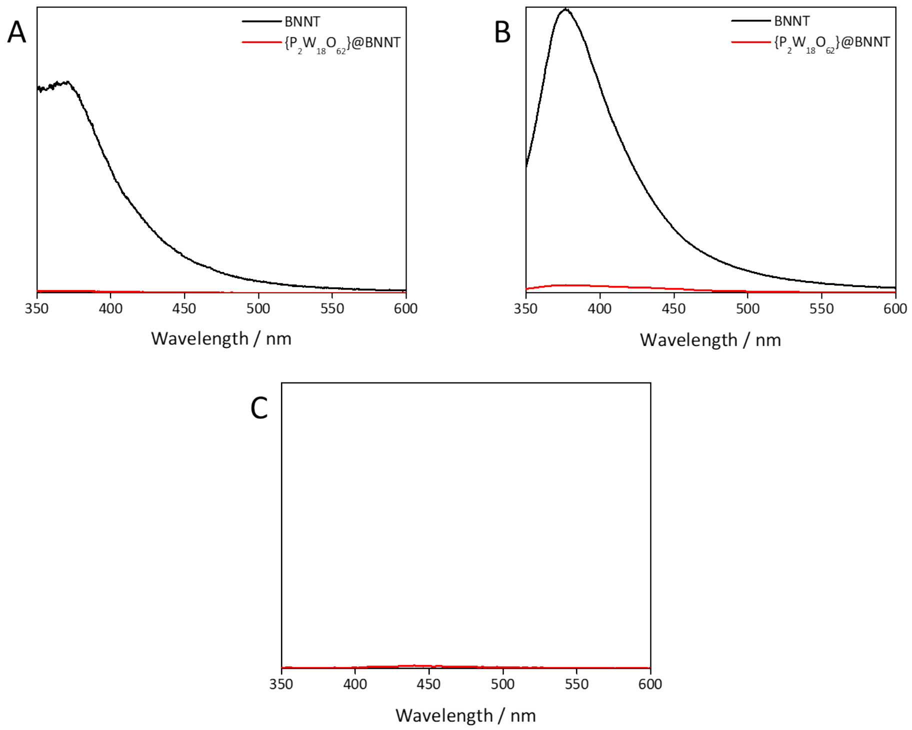

Figure S4. PL spectra of BNNTs and $\{P_2W_{18}O_{62}\}$@BNNT using excitation wavelengths of 250 nm (A) and 275 nm (B). (C) shows POM PL spectra using an excitation wavelength of 275 nm.

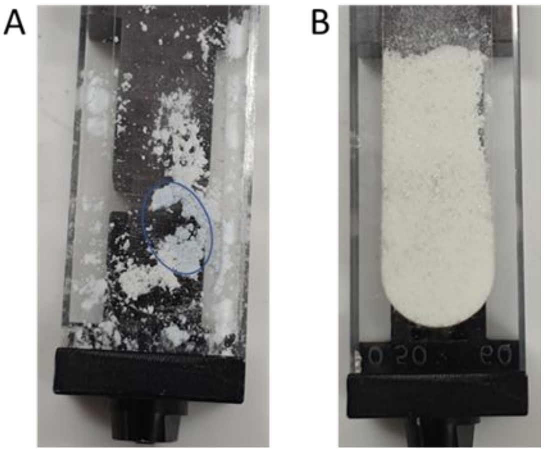

Figure S5. $\{P_2W_{18}O_{62}\}$@BNNT (A) and $K_6[P_2W_{18}O_{62}]$ (B) materials after PL spectroscopy measurements. Blue colouration (corresponding to the illumination spot) is visible in the $\{P_2W_{18}O_{62}\}$@BNNT material (blue circle).

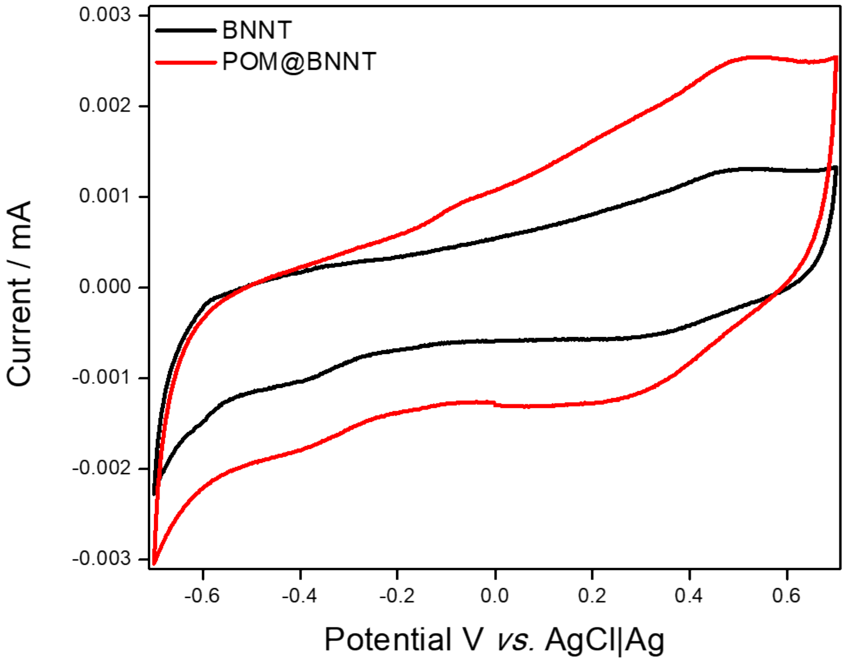

Figure S6. Cyclic voltammograms of thin films of BNNT (black trace) and POM@BNNT (red trace) recorded in 1M H_2SO_4 at a scan rate of 100 mV s^{-1} with a glassy carbon working electrode (film deposited from 10 mg/mL DMF suspension) AgCl|Cl reference electrode and platinum counter electrode. No faradaic current from the encapsulated POMs was observed, likely due to the insulating nature of the BNNTs.
